# Quantification of myocardial ^99m^Tc-labeled bisphosphonate uptake with cadmium zinc telluride camera in patients with transthyretin-related cardiac amyloidosis

**DOI:** 10.1186/s13550-019-0584-8

**Published:** 2019-12-23

**Authors:** Alain Manrique, David Dudoignon, Stéphanie Brun, Catherine N’Ganoa, Emmanuelle Cassol, Damien Legallois, Yoan Lavie-Badie, Denis Agostini, Olivier Lairez

**Affiliations:** 1 Department of Nuclear Medicine, Normandie Univ, UNICAEN, EA4650 SEILIRM, CHU de Caen, 14000 Caen, France; 2 GIP Cyceron, Campus Jules Horowitz, Boulevard Henri Becquerel, BP 5229, 14074 Caen, France; 3 Department of Nuclear Medicine, Toulouse University Hospital, Toulouse, France; 4 Medical School, Toulouse III Paul Sabatier University, Toulouse, France; 5 Department of Cardiology, Normandie Univ, UNICAEN, EA4650 SEILIRM, CHU de Caen, 14000 Caen, France; 6 Department of Cardiology, Rangueil University Hospital, Toulouse, France

**Keywords:** CZT SPECT, Cardiac amyloidosis, Bone scintigraphy, Planar imaging

## Abstract

**Purpose:**

We aimed to compare different methods for semi-quantitative analysis of cardiac retention of bone tracers in patients with cardiac transthyretin amyloidosis (ATTR).

**Methods:**

Data from 67 patients with ATTR who underwent both conventional whole-body scan and a CZT myocardial SPECT (DSPECT, Spectrum Dynamics) 3 h after injection of ^99m^Tc-labeled bone tracer were analyzed. Visual scoring of cardiac retention was performed on whole-body scan according to Perugini 4-point grading system from 0 (no uptake) to 3 (strong cardiac uptake with mild/absent bone uptake). A planar heart-to-background (H:B) ratio was calculated using whole-body scan (wb-H:B). CZT SPECT was quantified using three methods: planar H:B ratio calculated from anterior reprojection (ant-H:B), left anterior oblique reprojection (LAO-H:B), and 3D-H:B ratio calculated from transaxial slices as mean counts in a VOI encompassing the heart divided by background VOI in the contralateral lung. Interventricular septal thickness was obtained using echocardiography.

**Results:**

H:Bs obtained from planar and reprojected data were not statistically different (wb-H:B, 2.05 ± 0.64, ant-H:B, 1.97 ± 0.61, LAO-H:B, 2.06 ± 0.64, all *p* = ns). However, 3D-H:B was increased compared to planar H:Bs (3D-H:B, 4.06 ± 1.77, all *p* < 0.0001 vs. wb-H:B, ant-H:B, and LAO-H:B). Bland-Altman plots demonstrated that the difference between 3D and planar H:Bs increased with the mean value of myocardial uptake. 3D-H:B was best correlated to septal thickness (*r* = 0.45, *p* < 0.001). Finally, abnormal right ventricular uptake was associated with higher values of cardiac retention.

**Conclusion:**

3D semi-quantitative analysis of CZT SPECT optimized the assessment of ^99m^Tc-labeled bone tracer myocardial uptake in patients with cardiac amyloidosis.

## Introduction

Transthyretin amyloidosis (ATTR) is an important cause of heart failure with preserved ejection fraction (HFpEF) [1–3], especially in patients with increased wall thickness [4]. Cardiac planar radionuclide imaging using 99mTc-labeled bone-seeking radiopharmaceuticals may identify cardiac ATTR amyloid deposits, even in the early course of the disease, and is now widely used as a noninvasive diagnostic criterion in patients without detectable monoclonal protein [5, 6].

After the diagnosis, median survival ranges from 25 to 69 months depending on the transthyretin genotype (i.e., hereditary variant or wild-type ATTR) and the stage of the disease [7–9]. Medical therapy using Tafamidis meglumine, that binds to transthyretin and prevents amyloidogenesis, recently demonstrated a reduction in all-cause mortality and cardiovascular-related hospitalizations, offering new therapeutic perspectives in patients with ATTR cardiac amyloidosis. Several studies demonstrated that a high level of cardiac retention of bone radiopharmaceuticals is associated with a decreased survival in patients with ATTR cardiac amyloidosis [10, 11]. However, there is no standardized method for semi-quantitative assessment of cardiac uptake of bone tracers. Besides the visual assessment proposed by Perugini and colleagues [12] to identify patients with cardiac ATTR, several semi-quantitative methods have been proposed to evaluate patients prognosis, including heart to whole-body (H:WB) retention, heart to contralateral lung (H:CL) uptake ratio, and variation of regional left ventricular tracer uptake [10, 13–15].

In the context of a new era for medical therapy of ATTR cardiac amyloidosis, there is a need for quantitative approaches that would be useful for diagnosis, assessment of patient prognosis, and also therapeutic response. With the widespread use of cadmium zinc telluride (CZT)-based single-photon emission computed tomography (SPECT) camera, it is now feasible to propose a semi quantitative assessment of a cardiac tracer uptake using either 3D reconstructed acquisition or reprojected planar images [16, 17].

The aim of this study was to compare several indexes of cardiac uptake of bone tracers obtained with CZT SPECT in comparison to conventional planar bone scan acquisition in patients with cardiac ATTR.

## Material And methods

### Study population

Between October 2015 and March 2019, 97 consecutive patients with suspected cardiac ATTR were referred to the Nuclear Medicine department in two participating centers (Caen University Hospital and Toulouse University Hospital) for planar whole-body bone scintigraphy as part of routine diagnostic investigations. The need for supplemental tomographic acquisition was determined by the nuclear medicine physician on the basis of the results of whole-body scan. The final diagnostic of cardiac ATTR was based on the results of immunofixation electrophoresis of serum and urine, serum free light chain assay, and bone scintigraphy. Patient characteristics and echocardiography results were obtained from hospital records. The investigation conforms with the principles outlined in the Declaration of Helsinki. This retrospective study was approved by our institutional review board.

### Bone scintigraphy

Patients were scanned after intravenous injection of 10 MBq/kg of bisphosphonates ([^99m^Tc]TcDPD in Caen and [^99m^Tc]TcHMDP in Toulouse). Whole-body planar images were acquired 3 h after injection, followed by a SPECT acquisition.

Whole-body images were acquired using a conventional Anger camera (Symbia, Siemens, Erlangen, Germany) with low energy, high-resolution collimators and a scan speed of 10 cm/min. Cardiac retention was assessed by the semiquantitative visual score proposed by Perugini et al. [12] from 0 (no uptake) to 3 (uptake greater than bone, bone uptake attenuation, and soft tissue uptake). Additional tomographic acquisition was not performed in the case of Perugini score = 0.

The tomographic imaging was started with a 10-s prescan to help position the detectors on the cardiac area, followed by a 10-min list mode using a dedicated CZT cardiac SPECT camera (D-SPECT; Spectrum Dynamics, Biosensors, Caesarea, Israel) and a 10 % asymmetrical (− 7 to + 9 keV) energy windows centered on 140.5 keV. No scatter correction was performed, and reconstruction was performed using a dedicated workstation provided by the manufacturer. All acquisitions were gated at 16 intervals per cardiac cycle. Left ventricular volumes and ejection fraction were calculated using QGS software (Cedars-Sinai, Los Angeles, CA, USA). In addition to tomographic reconstruction, two planar equivalent images (planograms) were obtained as previously described [17] by projecting and summing all the elementary 2D images that shared the same angle onto one large field of view virtual plane in anterior and 45° left anterior oblique views.

### Heart-to-background ratio analysis

Different heart-to-background (H:B) uptake ratio were calculated using planar and SPECT imaging by drawing regions of interest (ROI) manually on the left ventricle and over a background region. Using planar conventional imaging, a ROI was manually drawn over the heart in the anterior view, and then copied and pasted over the contralateral chest, including soft tissue, ribs, and blood pool. On planograms, a circular ROI was drawn over the heart while the size of the background ROI was determined automatically on the *x* and *y* dimensions and positioned manually over the contralateral chest (in anterior view) or the mediastinum (in LAO view). Finally, in transaxial reconstructed SPECT images, an elliptic volume of interest (VOI) was drawn manually to encompass the heart and a background VOI was placed over the contralateral lung. As a result, four different H:B uptake ratios were generated: wb-H:B (from planar whole-body image), ant-H:B (from anterior planogram), LAO-H:B (from left anterior oblique planogram), and 3D-H:B (from transaxial slices).

### Statistical analysis

Paired data were compared using Student’s *t* test for paired samples, and unpaired data were compared using ANOVA. Correlations and concordance between quantitative variables were tested using linear regression analysis with Pearson’s correlation and Bland-Altman analysis. Multivariate analysis was performed using a linear model. A two-tailed *p* value ≤ 0.05 was considered statistically significant. Statistical analysis was performed using JMP® version 11.0 (SAS Institute Inc., Cary, NC).

## Results

### Study population

Among the 97 consecutive patients screened in two university hospital (CHU de Caen, *n* = 51, and CHU de Toulouse, *n* = 38), 3 were excluded due to a final diagnosis of AL amyloidosis, and 27 were excluded due to incomplete SPECT or whole-body dataset. Finally, SPECT and planar whole-body radionuclide imaging collected from 67 patients were reviewed. Patient characteristics are summarized in Table 1. The cohort was predominantly male (57/67 patients, 85%) with NYHA class II or class III symptoms. Patients received either [^99m^Tc]TcDPD (*n* = 37) or [^99m^Tc]TcHMDP (*n* = 30). These patients presented with cardiac hypertrophy and relatively preserved ejection fraction, and 15 patients (25%) were in NYHA class 0–I at the time of investigation.

**Table 1:** Patients characteristics

Age	81 ± 6
Gender, male, *n* (%)	57 (85)
NYHA class, *n* (%)	
0–I	17 (25)
II	18 (27)
III	20 (30)
IV	12 (18)
Echocardiography	
Septal thickness, mm	17 ± 4
LVEF, %	47 ± 14
Perugini classification, *n* (%)	
Grade 1	3 (5)
Grade 2	28 (42)
Grade 3	36 (53)
Heart-to-background ratio	
Whole-body	2.05 ± 0.64
SPECT anterior projection	1.97 ± 0.61
SPECT LAO projection	2.06 ± 0.64
SPECT 3D	4.06 ± 1.77
CZT SPECT	
LV EDV, ml	148 ± 55
LV ESV, ml	93 ± 50
LV EF, %	41 ± 15
Right ventricular uptake, *n* (%)	20 (30)

### Cardiac retention of bone radiopharmaceuticals

Whole-body planar images demonstrated an abnormal cardiac retention of bone radiopharmaceuticals, predominantly graded Perugini 2 and 3 (Table 1). All planar methods (whole-body and planograms) yielded H:B uptake ratios that were not statistically different. However, 3D-H:B ratio was dramatically increased compared to planar results in patients with Perugini grade 2 and grade 3 (Table 2). Multivariate analysis using linear model confirmed that H:B uptake ratio was significantly influenced by both the Perugini grade (*p* < 0.0001) and the calculation method (*p* < 0.0001).

**Table 2:** Heart-to-background uptake ratio according to the Perugini grade

Heart-to-background ratio	Perugini 1	Perugini 2	Perugini 3
Whole-body	1.45 ± 0.04*	1.65 ± 0.22^†/‡^	2.42 ± 0.67^‡^
SPECT anterior projection	1.51 ± 0.36^††^	1.63 ± 0.24^†/‡^	2.27 ± 0.67^‡^
SPECT LAO projection	1.24 ± 0.06*	1.77 ± 0.29^†/‡^	2.35 ± 0.70^‡^
SPECT 3D	1.96 ± 0.88*	3.07 ± 0.63^†^	5.01 ± 1.85

*p < 0.01. ^†^ *p* < 0.0001 and ^††^*p* < 0.05 vs. Perugini grade 3. ^‡^*p* < 0.0001 vs. SPECT 3D

**Fig. 1:**
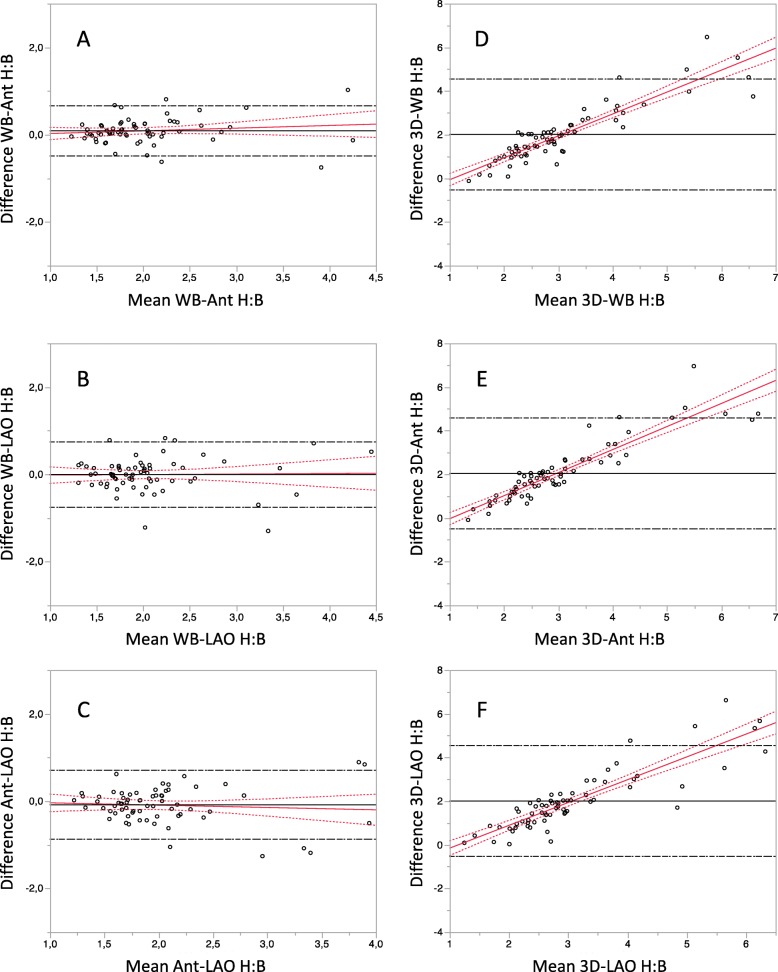
Bland-Altman analysis showing (1) a high agreement between all planar methods for the measurement of the heart-to-background (H:B) uptake ratio (**a**-**c**) and (2) an underestimation of the high H:B uptake ratio using planar methods as shown by the increased difference between 3D-H:B and planar H:B ratios for high H:B ratio values (**d**–**f**). WB, whole-body H:B; Ant, anterior planogram H:B; LAO, left anterior oblique planogram H:B

The correlation was high between all H:B ratios (Table 3). Bland-Altman analysis confirmed a high agreement between all planar methods (Fig. 1).

**Table 3:** Regression analysis and Pearson’s correlation between each individual method for heart-to-background (H:B) uptake ratio

*X*	*Y*	Regression	*r*	*p* value
WB-H:B	Ant-H:B	*Y* = 0.2396689 + 0.8404054*X	0.89	< 0.0001
WB-H:B	LAO-H:B	*Y* = 0.3885076 + 0.8136067*X	0.82	< 0.0001
WB-H:B	3D-H:B	*Y* = − 0.536663 + 2.2380835*X	0.82	< 0.0001
Ant-H:B	LAO-H:B	*Y* = 0.445274 + 0.8212357*X	0.78	< 0.0001
Ant-H:B	3D-H:B	*Y* = − 0.521782 + 2.3309197*X	0.80	< 0.0001
LAO-H:B	3D-H:B	*Y* = − 0.183172 + 2.0604316*X	0.74	< 0.0001

However, compared to planar methods, 3D analyses consistently showed higher values and in addition showed increasing differences with higher uptake values.

**Fig. 2:**
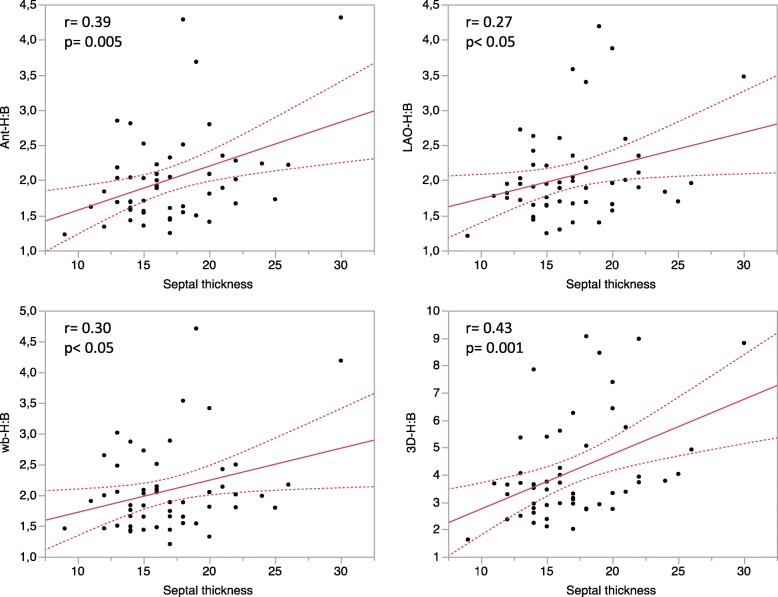
Correlation of the heart-to-background (H:B) uptake ratio to the septal wall thickness. WB, whole-body H:B; Ant, anterior planogram H:B; LAO, left anterior oblique planogram H:B

Cardiac retention of bone radiopharmaceuticals was correlated to septal wall thickness. As depicted in Fig. 2, the higher correlation between cardiac uptake and septal wall thickness was obtained when using 3D-H:B. Finally, patients with an abnormal right ventricular uptake of bone radiopharmaceutical on CZT SPECT imaging demonstrated higher H:B ratios compared to patients without right ventricular uptake (Table 4).

**Table 4:** Heart-to-background uptake ratio according to the right ventricular (RV) uptake

Heart-to-background ratio	No RV uptake	Positive RV uptake	*p* value
Whole-body	1.78 ± 0.41	2.17 ± 0.69	< 0.01
SPECT anterior projection	1.77 ± 0.31	2.03 ± 0.65	ns
SPECT LAO projection	1.95 ± 0.56	2.08 ± 0.68	< 0.05
SPECT 3D	3.46 ± 1.18	4.21 ± 1.82	< 0.05

## Discussion

The results of this study demonstrated that a 3D semi-quantitative analysis of CZT SPECT is feasible and increases the heart-to-background ratio of ^99m^Tc-labeled bone seeking tracer uptake compared to bidimensional methods, with a difference between the two methods increasing with the severity of the disease.

Cardiac ATTR is an underdiagnosed disease, more common than previously estimated [2], and that predominantly affects patients in their seventh and eighth decade of life [18]. Echocardiographic and cardiac magnetic resonance imaging patterns are non-specific, demonstrating a left ventricular hypertrophy that is frequently absent in the early phase. Advanced techniques using speckle-tracking imaging may reveal an apical sparing of longitudinal strain that enhances diagnostic accuracy but remain poorly investigated [19]. In addition to left ventricular hypertrophy, cardiac magnetic resonance imaging may show a late gadolinium enhancement, especially within the subendocardium, that increases diagnostic sensitivity [20]. On the other hand, it has been demonstrated that cardiac uptake of bone radiopharmaceuticals can early identify cardiac ATTR and help distinguish transthyretin from light chain cardiac amyloidosis [5, 11, 12, 21]. Consequently, bisphosphonate ([^99m^Tc]TcDPD, [^99m^Tc]PyP or [^99m^Tc]TcHMDP) scintigraphy is now a key feature in the diagnostic algorithm in patients with suspected cardiac ATTR [5, 22].

However, the visual assessment of cardiac uptake according to the widely used Perugini grading failed to predict patient prognosis [23]. Recent data pointed out that the intensity of cardiac uptake is a powerful predictor of poor cardiac outcome [10, 11]. Rapezzi et al. [11] demonstrated that a H:WB ratio of [^99m^Tc]TcDPD uptake > 7.5 was predictive of major cardiac adverse event. In this study, the calculation of the H:WB ratio required a correction for decay and scan speed and a subtraction of the urinary tract and bladder activity that are potential sources of errors. In a multicenter study, Castano et al. alternatively evaluated ^99m^Tc-PYP cardiac retention by the H:CL ratio of total counts using planar cardiac imaging and demonstrated an increased 5-year mortality in patients with H:CL ≥ 1.6 [10]. Several other indices have been proposed to quantify the amyloid burden using conventional Anger cameras, including heart/skull ratio, SPECT left ventricle/blood pool ratio, and heart/pelvis and heart/mediastinum ratio [13, 21, 24], demonstrating the lack of standardization of nuclear imaging procedures. In a recent study, Gallini et al. evaluated six different indices obtained from planar whole body imaging using conventional Anger camera. Using ROC curve analysis, the sensitivity of these indices ranged from 89 to 100%, with cutoff values ranging from 1.29 to 3.28. In this study, the heart/whole-body ratios were the most accurate in identifying cardiac amyloidosis [13].

Despite the widespread use of dedicated cardiac CZT cameras, this is the first study comparing the assessment of amyloid load using these cameras in comparison with conventional whole-body bone scan. Planar equivalent images, i.e., planograms, were obtained from SPECT acquisition as previously described [17]. The H:B ratio obtained from planograms were significantly lowers than those obtained from 3D images. The count statistics in heart and background regions in the planograms take into account the projection of every anatomic structure that binds the bone radiopharmaceutical, including the rib cage, the sternum, and the soft tissues [25]. Extra-cardiac uptake, including soft tissue involvement, is not constant and varies according to the stage of the disease [23]. On the other hand, the H:B ratio obtained from 3D reconstructed images reflect the heart retention normalized to the lung uptake, a metric that is not influenced by sternum, rib cage, or muscle uptake that are not included in VOIs. Interestingly, Glaudemans et al. [21] using a conventional Anger SPECT camera found an increased left ventricle-to-blood pool ratio in patients with ATTR and left ventricular hypertrophy that was further increased compared to H:WB ratio (4.6 vs. 2.9). Compared to these previous results, we found relatively higher H:B ratio. A possible explanation is the increased sensitivity of dedicated CZT cameras, with a linear count rate response compared to the non-linear response with a saturation at high count rate observed with conventional Anger cameras [26]. Accordingly, our results demonstrated that the underestimation of cardiac retention by planar techniques increases with the mean value of this retention as assessed by 3D CZT SPECT imaging. In addition, 3D H:B provided a good correlation to myocardial thickness, and 3D analysis was able to demonstrate a right ventricular involvement in 30% of the patients, a condition associated with an increased cardiac retention.

## Conclusion

The semi-quantitative analysis of 3D CZT SPECT optimized the assessment of bisphosphonate myocardial uptake compared to 2D methods in patients with cardiac amyloidosis.

## Data Availability

The datasets used and/or analyzed during the current study are available from the corresponding author on reasonable request
